# Transcriptomic and immunologic implications of the epithelial–mesenchymal transition model reveal a novel role of SFTA2 in prognosis of non-small-cell lung carcinoma

**DOI:** 10.3389/fgene.2022.911801

**Published:** 2022-08-26

**Authors:** Na Li, Zhanqiang Zhai, Yuanbiao Chen, Xiaofeng Li

**Affiliations:** ^1^ Department of Respiratory Medicine, The First Hospital of Jiaxing (the Affiliated Hospital of Jiaxing University), Zhejiang, China; ^2^ Department of Thoracic Disease Center, Zhejiang Rongjun Hospital, Zhejiang, China; ^3^ Affiliated Hospital of Youjiang Medical University for Nationalities, Baise, China

**Keywords:** epithelial–mesenchymal transition (EMT), non-small-cell lung cancer (NSCLC), SFTA2, prognosis, tumor microenvironment

## Abstract

Non-small-cell lung cancer (NSCLC) is the second most common cancer worldwide, and most deaths are associated with epithelial–mesenchymal transition (EMT). Therefore, this study aimed to explore the role of EMT-related transcriptomic profiles in NSCLC and the effect of EMT-based signatures on clinical diagnosis, prognosis, and treatment responses for patients with NSCLC. After integrating the transcriptomics and clinicopathological data, we first constructed EMT clusters (C1 and C2) using machine learning algorithms, found the significant relationship between EMT clusters and survival outcomes, and then explored the impact of EMT clusters on the tumor heterogeneity, drug efficiency, and immune microenvironment of NSCLC. Prominently, differential-enriched tumor-infiltrated lymphocytes were found between EMT clusters, especially the macrophages and monocyte. Next, we identified the most significantly down-regulated gene SFTA2 in the EMT clusters C2 with poor prognosis. Using RT-qPCR and RNA-seq data from the public database, we found prominently elevated SFTA2 expression in NSCLC tissues compared with normal lung tissues, and the tumor suppressor role of SFTA2 in 82 Chinese patients with NSCLC. After Cox regression and survival analysis, we demonstrated that higher SFTA2 expression in tumor samples significantly predicts favorable prognosis of NSCLC based on multiple independent cohorts. In addition, the prognostic value of SFTA2 expression differs for patients with lung adenocarcinoma and squamous cell carcinoma. In conclusion, this study demonstrated that the EMT process is involved in the malignant progression and the constructed EMT clusters exerted significant predictive drug resistance and prognostic value for NSCLC patients. In addition, we first identified the high tumoral expression of SFTA2 correlated with better prognosis and could serve as a predictive biomarker for outcomes and treatment response of NSCLC patients.

## Introduction

Lung cancer is the second most common cancer worldwide (Akram et al., 2018). Non-small-cell lung cancer (NSCLC) accounts for 85% of all lung cancer cases, and the global incidence of NSCLC is estimated to be approximately 2 million new cases per year (Zhou et al., 2021). Most patients are diagnosed at an advanced stage of the cancer (Kwan and Chowdhury, 2021), suffering from a poor prognosis and high risk of death. With poor efficacy of monotherapy, targeted therapy with specific inhibitors is now the most promising first-line cancer therapy on the market (Osmani et al., 2018). Numerous studies related to the discovery of candidate biomarkers for lung cancer have been published (Gong et al., 2020). However, none of these biomarkers are widely used in clinical practices, and the function of these candidate signatures still lacks validation in well-designed large-scale studies (Rybarczyk-Kasiuchnicz et al., 2021).

The vast majority of cancer-related deaths, such as hepatocellular carcinoma and breast cancer (Saxena et al., 2021; Gao et al., 2022), are due to metastatic spread of cancer cells, a process aided by epithelial–mesenchymal transition (EMT) (Atay, 2020; Kitz et al., 2021). EMT refers to the biological process in which epithelial cells are transformed into cells with a mesenchymal phenotype through specific programs (Zhang et al., 2022). In the process of EMT, the polarity of epithelial cells is lost, contact with surrounding cells and stromal cells is reduced, interaction between cells is reduced, and ability of cell migration and motility is enhanced (Ramesh et al., 2020). At the same time, the cell phenotype changes and the expression level of E-cadherin decreased, resulting in reduced cell adhesion, allowing cells to acquire the characteristics of easy invasion and metastasis (Wu et al., 2020).

EMT also plays a very important role in the malignant evolution and migration capacities of tumors (Pastushenko and Blanpain, 2019; Xu et al., 2020a; Liu et al., 2021). EMT enables tumor cells in the primary site to acquire the ability to move and invade and also enables tumor cells to escape from apoptosis induced by certain factors, increasing their antiapoptotic ability (Singh et al., 2018; Bakir et al., 2020). Tumor cells exuding from blood vessels or lymphatics could revert to an epithelial state through mesenchymal–epithelial transition and then proliferate to form large and even macroscopic secondary tumors. For example, transient inhibition or overexpression of fatty acid synthase significantly regulated kidney cancer cells proliferation and migration by regulating the EMT process (Xu et al., 2020b). Many EMT-related signaling pathways and transcription factors have been confirmed to be involved in the drug resistance of NSCLC (Mahmood et al., 2017). Inhibition of tumor EMT signaling can delay tumor drug resistance, thereby improving efficacy and reducing toxicity (Chae et al., 2018; Wu et al., 2021). Therefore, this study aimed to investigate the effect of EMT on NSCLC and the EMT-related hub gene. We speculate that the expression of the EMT pathway is related to the prognosis and clinical treatments in NSCLC patients.

## Methods

### Patients and tissue samples from online databases

Raw counts of RNA-sequencing data and corresponding clinical information from 1,000 NSCLC patients were obtained from The Cancer Genome Atlas (TCGA) database (https://portal.gdc.cancer.gov/) as a training cohort. After we excluded patients with unclear clinicopathological information and missing overall survival information, we finally included 1,000 NSCLC patients with present survival, clinicopathological information, and transcriptomic data. In addition, 1,144 NSCLC patients with clinical follow-up information were enrolled in GSE19188, GSE30219, GSE29013, GSE50081, and GSE37745 cohorts from the Gene Expression Omnibus (GEO) data sets (https://www.ncbi.nlm.nih.gov/geo/) as testing cohorts. In addition, we enrolled 82 NSCLC patients with available survival and clinicopathological data from tissue bank of the Affiliated Hospital of Youjiang Medical University for Nationalities (Youjiang cohort, Youjiang, China) as a validation cohort.

### Establishment of EMT clusters for patients with NSCLC from the TCGA database

Among 1,000 patients with NSCLC from the TCGA database, we integrated the level 3 RNA-seq data, extracted expression profiles of EMT process, and collected corresponding clinical and pathological data. Performing a consistency analysis using “ConsensusClusterPlus” and “pheatmap” R package, we observed the best unsupervised groups (less than 6) and performed clustering heat maps according to the progression-free survival. The gene expression heatmap retains genes with SD > 0.1. If the number of input genes is more than 1,000, it will extract the top 25% genes after sorting the SD.

### Analysis of tumor immune microenvironment and drug sensitivity of EMT clusters

We obtained a variety of immune infiltrating lymphocytes with different degrees of infiltration using the CIBERSORT algorithm (https://cibersort.stanford.edu) (Rusk, 2019). The differentially infiltrated lymphocytes were evaluated using the Wilcoxon rank sum test. The Tumor Immune Dysfunction and Exclusion (TIDE) and the semi-inhibitory concentration (IC50) tests were used to detect tumor heterogeneity between the two groups (Yang et al., 2013; Jiang et al., 2018).

### Expression validation of tumor and normal tissues by real-time quantitative PCR (RT-PCR)

A TissueScan^®^ qPCR cDNA array analysis (Origene, Rockville, MD, United States) was performed on 48 human tissues using SFTA2-specific primers, according to the manufacturer’s protocol. RNA was extracted from lung cancer and adjacent normal lung tissues, and a SFTA2 q-PCR analysis was performed with normalization for GAPDH. Primers used were SFTA2 forward: GGA​GTC​TTT​TCT​GAC​AAA​TTC​CTC and reverse: GGT​GTT​GAG​ATC​TTG​CAT​GGT​GG.

### Survival analysis

The Kaplan–Meier method was used to assess the significance of progression-free survival (PFS) and overall survival (OS) by using the Kaplan–Meier plotter (http://kmplot.com/analysis/index). Univariate and multivariate Cox regression analyses were performed to analyze the impact of SFTA2 expression, age, gender, and pathological TNM stages on overall survival. The forest was used to show the *p*-value, HR, and 95% CI of each variable through the “forestplot” R package.

### Differential expression analysis and functional enrichment analysis

To illustrate the statistically significance of differential expressed genes (DEGs) between EMT clusters, the “Limma” R package was used to compare the differences among NSCLC samples with threshold as follows: |Log_2_ (fold change)| > 1 and *p*-value < 0.05. Functional enrichment of all DEGs was performed using the KEGG database in a bubble chart.

### Statistics analysis

All statistical analyses were performed on R software and GraphPad Prism (Version 8.0). Group comparisons were determined by two-tailed *t* test and one-way ANOVA. Spearman’s correlation analysis was applied to determine significant correlation between linear variables. A survival analysis was visualized using the Kaplan–Meier curves. A univariate Cox regression analysis was performed using the R package “survival”. All tests with *p*-values < 0.05 were considered statistically significance.

## Results

### Construction of EMT clusters using machine learning algorithms

**FIGURE 1:**
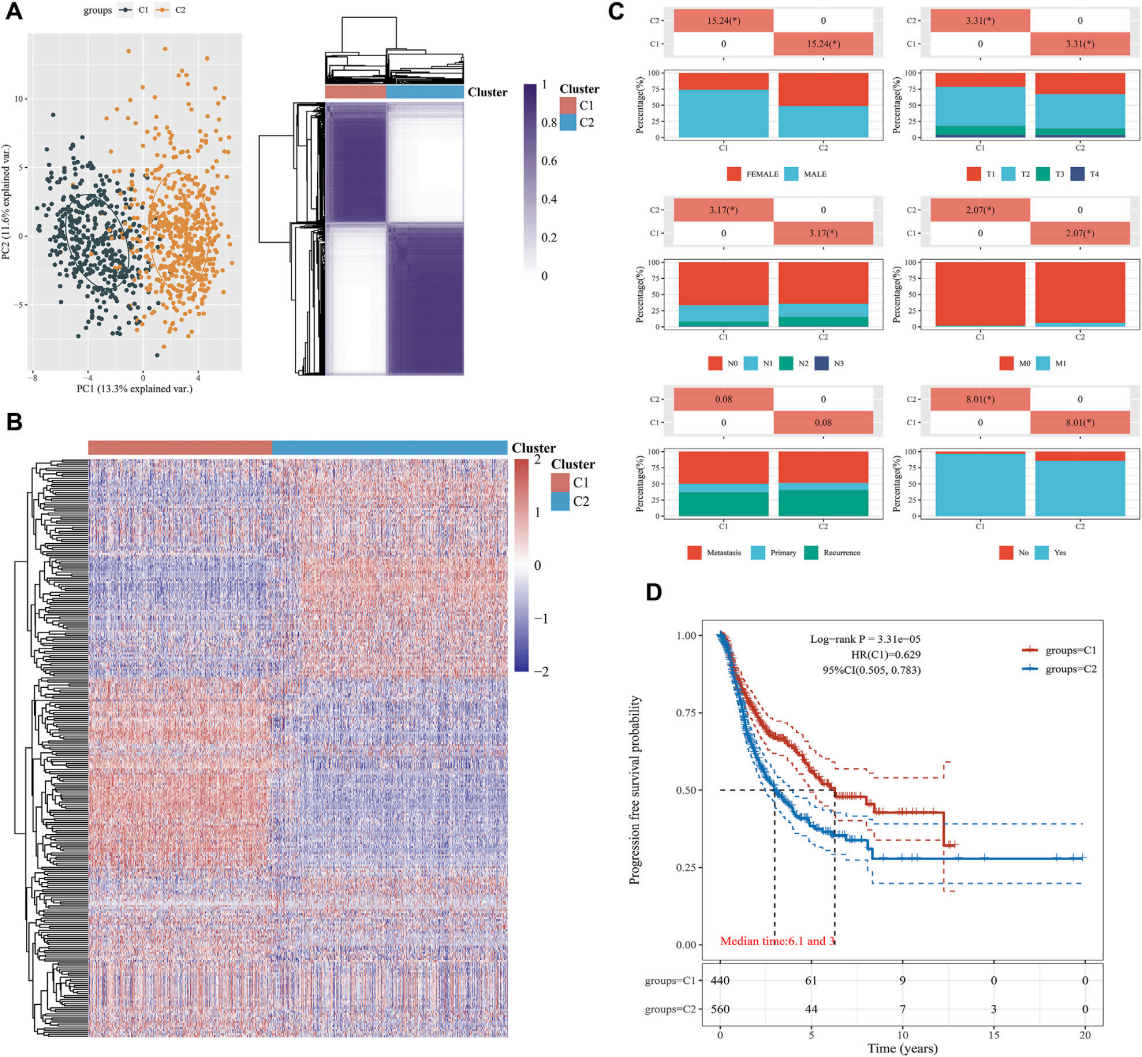
Construction of EMT clusters (C1 and C2) using machine learning algorithms. **(A)** The principal component analysis (PCA) was applied to explore differences in the expressed genes between the two groups (C1 and C2). Unsupervised clustering was used to classify NSCLC patients into groups (C1 and C2). **(B)** The heatmap of consistent clustering results shows the expression of EMT in each sample in C1 and C2 groups. Red represents high expression and blue represents low expression. **(C)** We investigated the distribution of some clinical characteristics in the two groups and analyzed significant *p*-values by using the chi-square test. **(D)** Kaplan–Meier survival curve analysis using median patient samples.

Based on 1,000 patients with NSCLC from the TCGA cohort, with unsupervised clustering, we prominently divided the samples into EMT Cluster1 and EMT Cluster 2, according to the EMT activity of NSCLC samples (Figures 1A,B). In order to further compare the differences between C1 group and C2, we selected some clinical and pathological factors, such as gender, race, TNM stage, and smoking status (Figure 1C). As shown in Table 1, there were significantly increased samples of progression, female, advanced pathological TNM stages, and non-smoking in EMT C2. Interestingly, we found more patients with smoking history in C2 than those in C1, suggesting that smoking is not significantly related to the EMT pathway in NSCLC patients.

**TABLE 1:** Clinical and pathological features according to the EMT clusters.

Clinical and pathological factors		EMT C1	EMT C2	*p*-value
Progression status	Progression	127	229	<0.001*
	Progression free	318	340	
	Mean (SD)	67.6 (8.5)	65.2 (9.9)	
Sex	Female	115	291	**<0.001**
	Male	330	278	
Race	Asian	6	10	0.784
	Black	31	51	
	White	305	431	
	American Indian	—	1	
pT stage	T1	41	75	**0.005**
	T1a	20	51	
	T1b	34	61	
	T2	155	184	
	T2a	82	87	
	T2b	33	28	
	T3	61	57	
	T4	19	23	
	TX	—	3	
pN stage	N0	292	357	**0.001**
	N1	113	113	
	N2	31	83	
	N3	4	3	
	NX	5	12	
pM stage	M0	356	399	**0.003**
	M1	5	18	
	M1a	1	2	
	M1b	1	5	
	MX	78	141	
pTNM stage	I	3	5	**<0.001**
	IA	75	145	
	IB	147	143	
	II	3	1	
	IIA	58	57	
	IIB	82	82	
	III	2	1	
	IIIA	51	85	
	IIIB	15	14	
	IV	7	26	
Metastasis status	Metastasis	34	62	0.546
	Metastasis: primary	2	1	
	Metastasis: recurrence	2	9	
	Primary	9	14	
	Recurrence	25	52	
Smoking history	Non-smoking	14	78	**<0.001**
	Smoking	421	475	
Radioation history	Non-radiation	132	148	0.928
	Radiation	14	14	

*p value <0.05 are marked in bold.

Moreover, a progression-free survival analysis showed that the prognosis of the group with EMT C1 was markedly better (log-rank *p* = 3.31e-05, HR = 0.629), with median time of 6.1 month in C1 and 3.0 month in C2 (Figure 1D). In addition, we then evaluated the differential expression of traditional EMT markers, including CDH1, CDH2, CLDN1, SNAI1, SNAI2, TGFB1, TWIST1, VIM, ZEB1, and ZEB2, between the EMT clusters. The findings suggested significant elevated expression of CDH1, CLND1, SNAI2, TGFB1, and TWIST1 in the EMT Cluster 1 and significant increased expression of CDH2, SNAI1, VIM, ZEB1, and ZEB2 in EMT Cluster 2 (Supplementary Figure S1). Overall, EMT clusters significantly correlated with clinicopathological indicators and predicts PFS in 1000 NSCLC patients from the TCGA database.

### EMT clusters divide differential tumor-infiltrated lymphocytes and immune microenvironment

**FIGURE 2:**
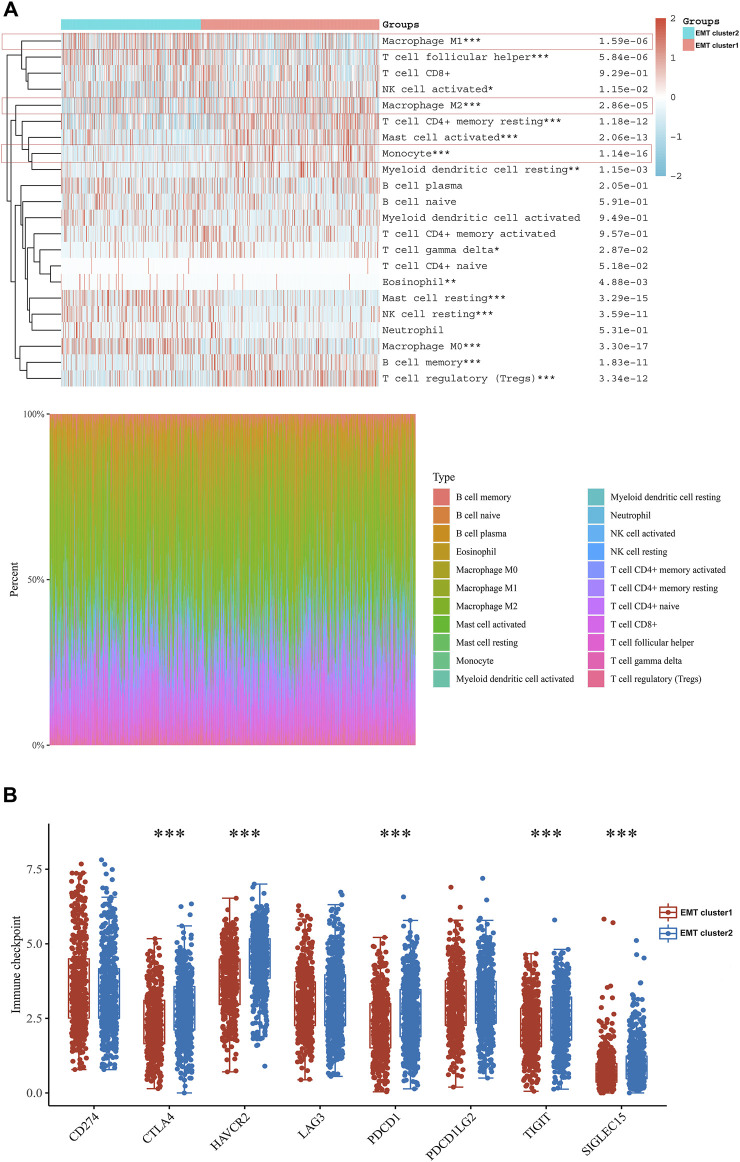
EMT clusters divide differential tumor-infiltrated lymphocytes and immune microenvironment. **(A)** We found the connection between different immune cells and EMT. Red represents positive correlation while blue represents negative correlation. **(B)** Expression distribution of immune checkpoint molecules in C1 and C2 groups.

After understanding the differential activity of the EMT process in EMT clusters, we focused on the impact of EMT clusters on tumor immune microenvironment and clinical treatment. We screened the aggregation of important immune cells in both groups and found that activity of macrophage M1 cells and resting mast cells were significantly higher in C2. However, in the C1 group, there were significantly enriched macrophage M2, monocytes, and activated mast cell (Figure 2A). Since M1 mainly secretes proinflammatory factors and M2 expression inhibits inflammatory factors, which plays a role in inhibiting inflammatory response and tissue repair, the better prognosis of the EMT C1 group may be related to the immune-excluded microenvironment. Though the C1 has a better prognosis and a more positive immune response, we compared and analyzed immune checkpoints and found that the C2 had higher expression of immune checkpoints, such as CTLA2, HAVCR2, LAG3, PDCD1, TIGIT, and SIGLEC15 (Figure 2B). These finding suggested that immunotherapy could be a potential treatment selection for NSCLC patients in the EMT C2 group.

### Intratumoral heterogeneity and drug sensitivity of EMT clusters in NSCLC

**FIGURE 3:**
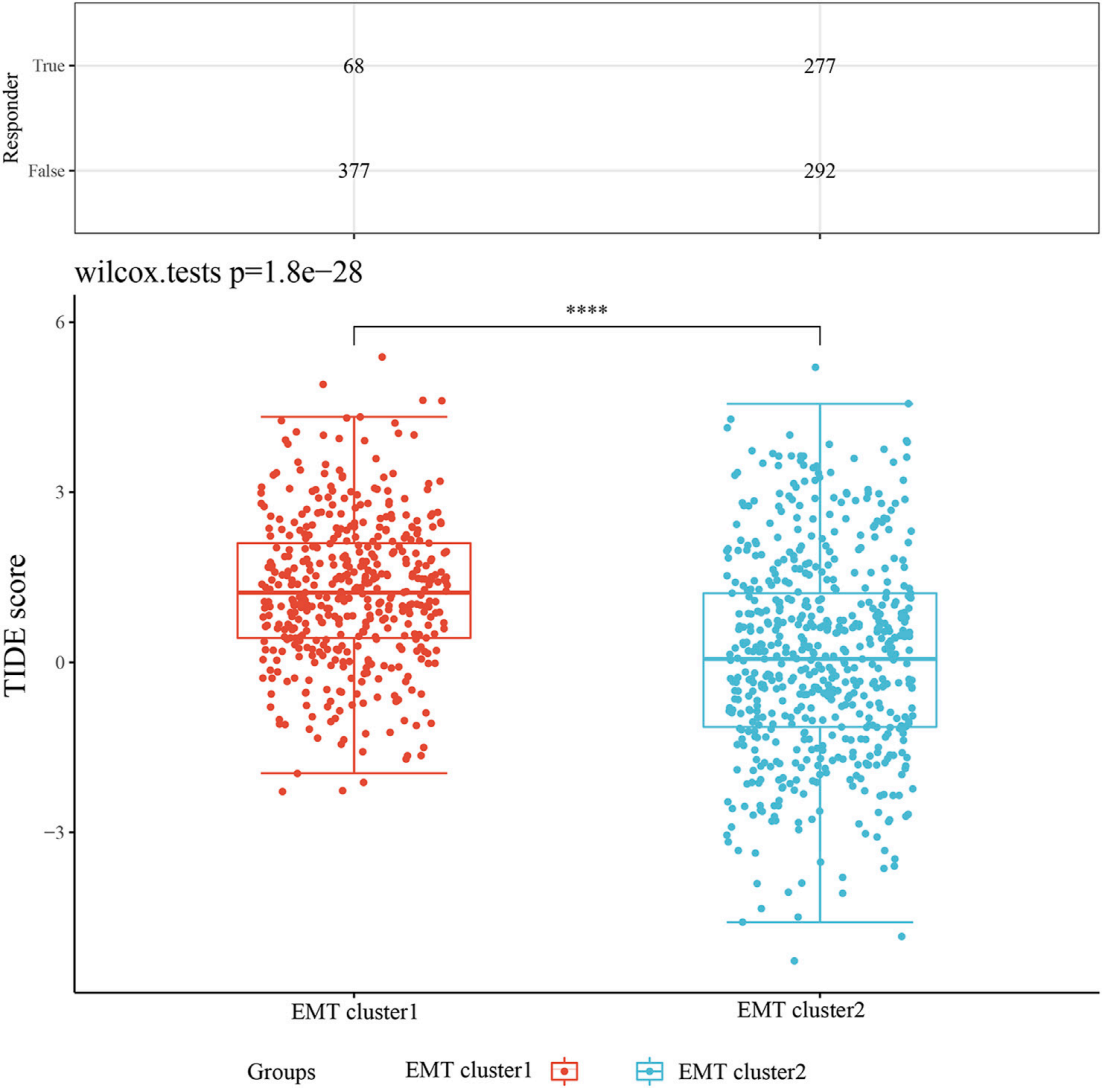
Intratumoral heterogeneity of NSCLC. The TIDE test was used to predict response to immune checkpoint inhibition therapy.

The TIDE score showed that the score of C2 was significantly lower than that in the C1 group, indicating that the EMT cluster 2 samples had lower heterogeneity and all tumor cells could have strong sensitivity to the given treatment (Figure 3). In addition, patients in EMT cluster 2 could receive favorable responses to immune checkpoint inhibitors and easier to obtain precise treatment strategies. Then we evaluated the effect of targeted therapy (first-generation EGFR tyrosine kinase inhibitors) and chemotherapy using the semi-inhibitory concentration (IC50) method, an important indicator for evaluating drug efficacy or sample treatment response (version cgp 2016). As shown in Supplementary Figure 2, the results indicated that the IC50 values of EGFR tyrosine kinase inhibitors gefitinib, erlotinib, and cisplatin in EMT Cluster 1 were significantly higher lower than EMT Cluster 2 (*p* < 0.0001), revealing that NSCLC patients in EMT Cluster one received better clinical responses to tyrosine kinase inhibitors or chemotherapy.

### Function enrichment analysis of EMT clusters and the identification of tumor-suppressor SFTA2

**FIGURE 4:**
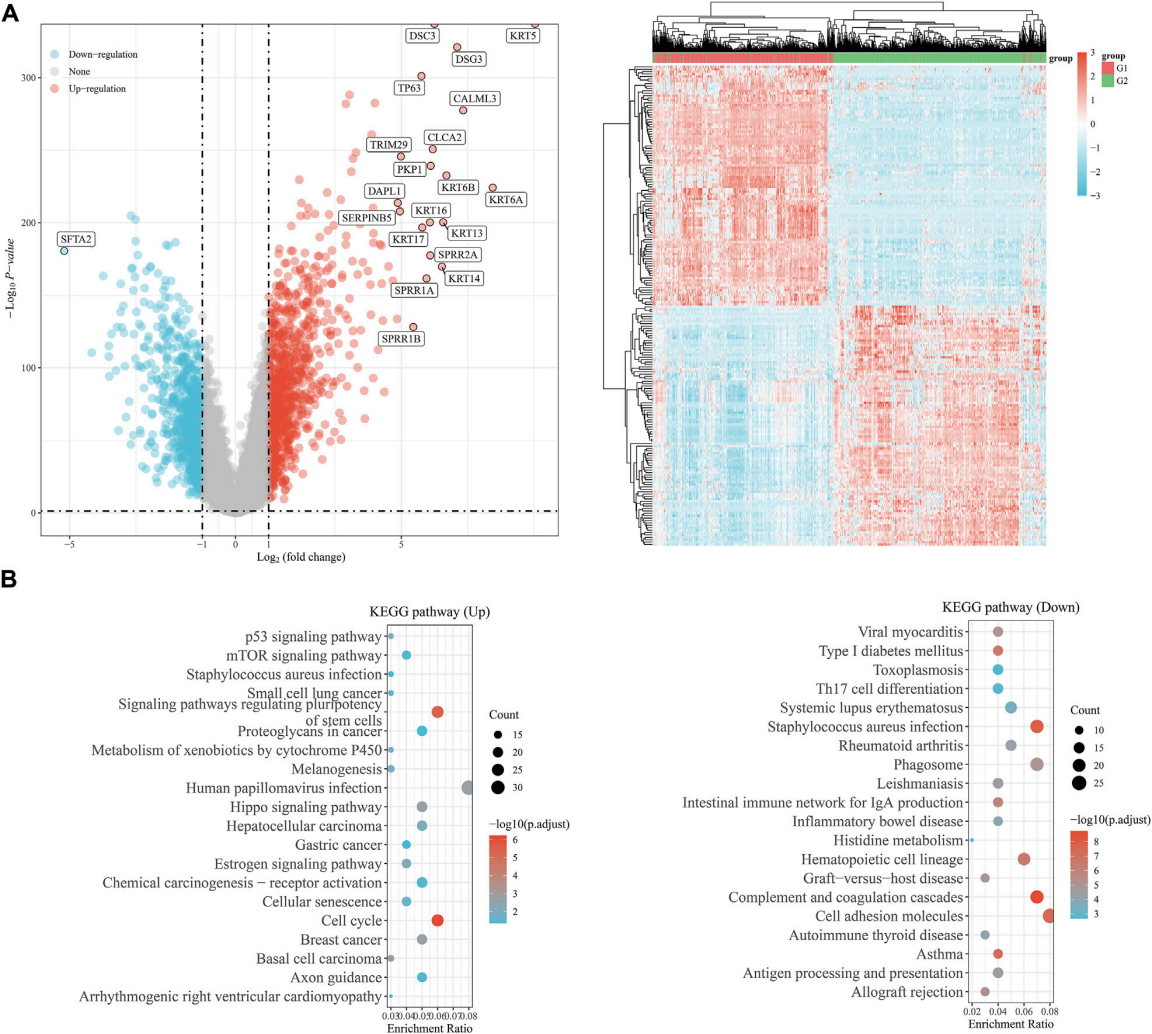
Function enrichment analysis of EMT clusters and the identification of tumor-suppressor SFTA2. **(A)** The graphs show the differentially expressed genes in the C1 and C2 groups, with up-regulated genes in red and down-regulated genes in blue. **(B)** We performed functional enrichment analysis to show the pathways involved in differentially up-regulated and down-regulated genes in the KEGG database.

Comparing the differential transcriptional profiles of EMT clusters, we listed down-regulated and up-regulated DEGs in EMT C2 (Supplementary Table S1). The most significantly down-regulated gene is SFTA2, a tumor suppressor gene, and the significantly up-regulated genes included KRT5, CLCA2, TP63, and so on in the volcano and clustering plot (Figure 4A). A functional enrichment analysis with samples from the KEGG database showed that the up-regulated genes were mainly involved in signaling pathways regulating pluripotency of stem cells and the cell cycle, while the down-regulated genes were mainly involved in *Staphylococcus aureus* infection, complement and coagulation cascades, and cell adhesion molecules pathway (Figure 4B).

### Potential implications of SFTA2 expression in outcomes of NSCLC

Furthermore, we compared the expression level of SFTA2 in tumor and normal tissues. To further illustrate the accuracy and reliability of the biomarker, we analyzed the expression level of the SFTA2 in human organs, and the results showed that the SFTA2 expression was most significantly expressed in lungs, followed by the esophagus (Supplementary Figures S3A,B).

**FIGURE 5:**
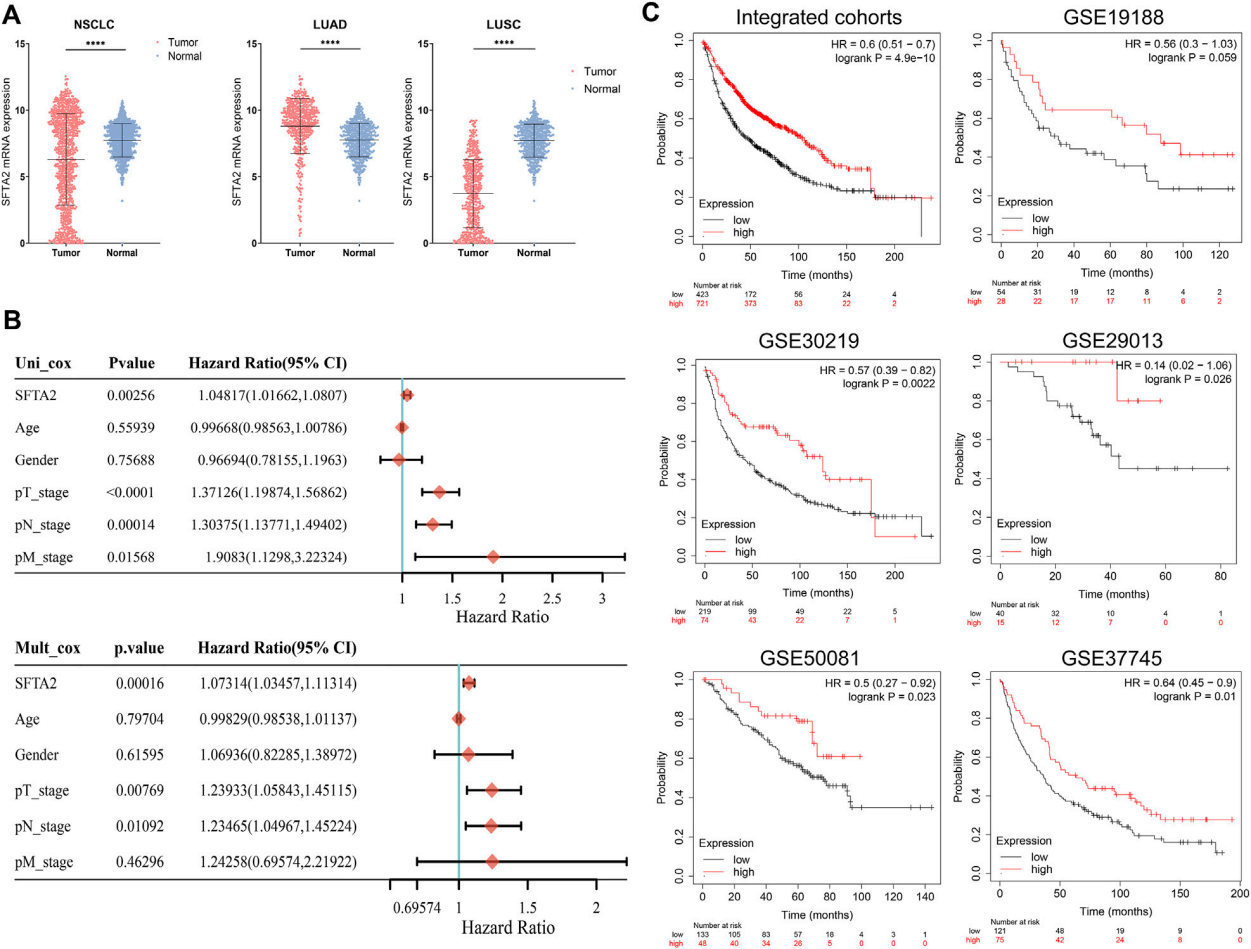
Potential implications of SFTA2 expression in outcomes of NSCLC. **(A)** We compared the expression of SFTA2 in normal and tumor tissues in NSCLC and subgroups of LUAD and LUSC. **(B)** We performed univariate and multivariate regression analyses to find the factors related to the survival of NSCLC patients. **(C)** We utilized independent cohort samples to study the relationship between SFTA2 expression and survival prognosis.

We then found that the expression of SFAT2 was significantly lower in tumor than that in normal tissues in NSCLC and squamous cell carcinoma (LUSC) cohorts, whereas the expression in adenocarcinoma (LUAD) was lower in normal tissue (Figure 5A). Table 2 shows the association of SFTA2 with clinicopathological factors, with increased samples of progression, female, non-white, advanced pTNM stage, and smoking in the SFTA2 high expression group. Next, we performed univariate and multivariate Cox regression analyses enrolling clinical and pathological indicators. The results suggested that the T stage, N stage, and SFTA2 were significantly associated with patient prognosis, indicating that SFTA2 could be a valuable independent biomarker of NSCLC (Figure 5B). Finally, with multiple independent cohorts and integrated cohort from the GEO database, we found that patients in the high SFTA2 expression group experienced better outcomes than that of SFTA2 low expression in cohorts like GSE30219 (*n* = 293, *p* = 0.0022, HR = 0.57), GSE33745 (*n* = 196, *p* = 0.01, HR = 0.64), GSE50081 (*n* = 181, *p* = 0.023, HR = 0.5), and GSE19188 (*n* = 82, *p* = 0.059, HR = 0.56) (Figure 5C).

**TABLE 2:** Clinical and pathological features according to the differential SFTA2 expression in 1,000 NSCLC patients from the TCGA database.

		Low SFTA2 expression	High SFTA2 expression	*p*-value
Progression status	Progression	195	161	0.030*
	Progression free	312	346	
	Mean (SD)	65.6 (9.6)	66.9 (9.1)	
Sex	Female	271	135	**<0.001**
	Male	236	372	
Race	American Indian	1		0.955
	Asian	8	8	
	Black	44	38	
	White	385	351	
pT stage	T1	72	44	**<0.001**
	T1a	50	21	
	T1b	51	44	
	T2	160	179	
	T2a	79	90	
	T2b	26	35	
	T3	47	71	
	T4	19	23	
	TX	3		
pN stage	N0	323	326	**<0.001**
	N1	93	133	
	N2	72	42	
	N3	5	2	
	NX	13	4	
pM stage	M0	351	404	**0.003**
	M1	16	7	
	M1a	2	1	
	M1b	3	3	
	MX	131	88	
pTNM stage	I	5	3	**<0.001**
	IA	136	84	
	IB	127	163	
	II	1	3	
	IIA	53	62	
	IIB	68	96	
	IIIA	72	64	
	IIIB	15	14	
	IV	22	11	
	III		3	
Metastasis status	Metastasis	59	37	0.865
	Metastasis: primary	1	2	
	Metastasis: recurrence	7	4	
Smoking history	Non-smoking	70	22	**<0.001**
	Smoking	422	474	
Radioation history	Non-radiation	143	137	**<0.001**
	Radiation	12	16	

*p value <0.05 are marked in bold.

### Expression and prognostic validation of SFTA2 expression in outcomes of NSCLC patients from the Chinese real-world cohort

**FIGURE 6:**
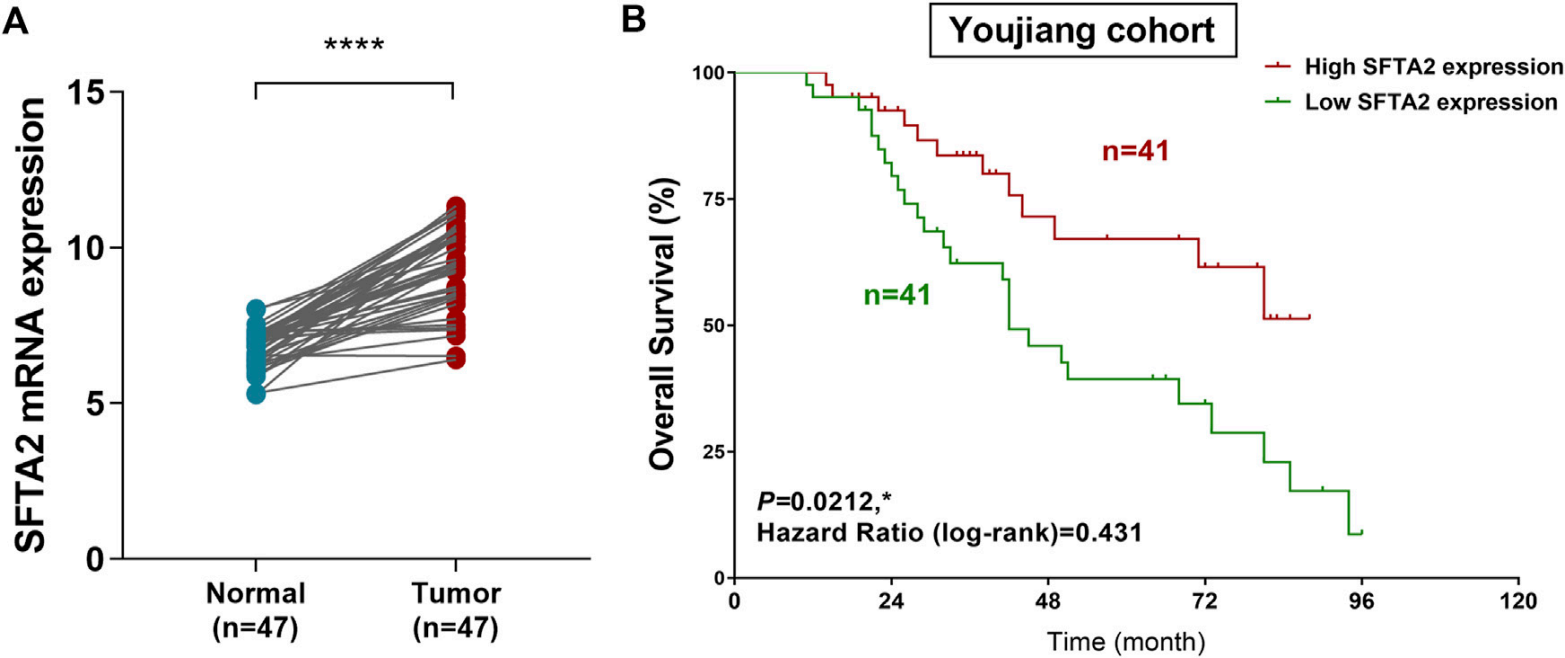
Expression and prognostic validation of SFTA2 expression in outcomes of NSCLC patients from the real-world cohort. **(A)** We compared the expression of SFTA2 in normal tissues (*n* = 47) and NSCLC (*n* = 47) tissues using Student’s t test. **(B)** Kaplan–Meier survival analysis and log-rank test reveal the prognostic value of SFTA2 expression in 82 patients from the Jiaxing cohort.

Next, we assessed mRNA expression of STFA2 in NSCLC tissues and normal tissues. The results validated our previous findings and showed significantly elevated expression level of SFTA2 in 47 paired tumor compared with normal tissues (*p* < 0.001) (Figure 6A). Then a total of 82 patients from the Youjiang cohort were enrolled in this study to validate our hypothesis. The clinical and pathological baseline data for patients with NSCLC in line with SFTA2 expression is shown in Supplementary Table S2. We found that SFTA2 serves as a significant tumor suppressor gene and could significantly predict prognosis for patients with lung adenocarcinoma (Figure 6B). The Kaplan–Meier survival analysis and the log-rank test indicated that low expression of SFTA2 significantly predicted poor OS for 82 Chinese patients with NSCLC from the real-world Jiaxing cohort (*p* = 0.0212, HR = 0.431). In addition, for patients with lung adenocarcinoma from the TCGA database, we found that decreased SFTA2 expression was not markedly associated with PFS (*p* = 0.059) while significantly predicted poorer OS (*p* = 0.0066). For patients with lung squamous cell carcinoma, elevated SFTA2 expression was not markedly associated with PFS (*p* = 0.310) while significantly predicted poorer OS (*p* = 0.01) (Supplementary Figure S4)

## Discussion

As one of the most common cancers in the world, lung cancer is characterized by late diagnosis, poor prognosis, and high mortality (Dundr et al., 2021; Zhao et al., 2022; Zheng et al., 2022). NSCLC is more common compared to small-cell lung cancer (SCLC), accounting for 85% of lung cancer patients (Fintelmann et al., 2015). Since there is no cure for NSCLC, the best supportive treatment is usually used for palliative treatment (NSCLC Meta-Analyses Collaborative Group, 2008). In NSCLC, driver mutations that activate the EGFR represent the most common actionable therapeutic alteration. EGFR tyrosine kinase inhibitors (TKIs) are the standard of care for first-line treatment of advanced or metastatic EGFR-mutant NSCLC. Early-stage treatment modalities include first-generation (1G; erlotinib, gefitinib), second-generation (2G; afatinib, dacomitinib), or third-generation (3G; osimertinib) EGFR TKIs, alone or in combination with other therapies. However, despite a high response rate of 80%, resistance inevitably develops after a median of 10–17 months (1–4 months) (Zhong et al., 2013).

The most common mechanism of resistance to 1G and 2G EGFR TKIs is the EGFR T790M “gatekeeper” mutation, which occurs in 50%–60% of patients (Yang et al., 2022a). Osimertinib is highly selective for T790M and was originally developed and approved for the treatment of T790M-positive (T790M+) resistance (Yang et al., 2022b). The mechanisms of resistance in the remaining patients, as well as those treated with 3G EGFR TKIs, are thought to be diverse and include activation of alternative signaling pathways, such as MET or HER2 amplification, or phenotypic changes of the EMT process, which is also a key cellular phenomenon involved in tumor metastasis and progression (Erin et al., 2020; Vokes et al., 2022). Overall, T790M-negative (T790M−) EGFR TKIs resistance patients remain a population with significant precision medical need.

It has been revealed that the EMT pathway and related transcription factors have been confirmed to be involved in cancer progression and resistance of EGFR TKIs NSCLC patients (Li et al., 2018; Meyer-Schaller et al., 2019). Therefore, we first divided the patients into two groups: C1 and C2. The study found that the difference between the EMT C1 and C2 groups was significant and was related to factors such as gender and clinical T and N stages. More importantly, we are the first to discover that prognosis of NSCLC patients with significantly associated with EMT. Since studies have shown that EMT cells can promote immune exclusion and deviation (Romeo et al., 2019). We then studied the immune cells related to EMT and the effects of EMT on the immune checkpoints. It was found that there were more active immune cells in the EMT C1 group, corresponding to the better prognosis of patients, while in the EMT C2 group, the expression of immune checkpoints was high, indicating that these patients might have better effect of receiving specific target immunotherapy.

Previous studies have interrogated the transcriptional profiles of TKI-resistant tumors and have suggested fundamental transcriptional differences in T790M+ and T790M− resistance mechanisms. Notably, the expression of lung adenocarcinoma marker genes such as SFTA2 and SFTA3 was widespread and almost completely lost in cancer cells of T790M− tumors (Kim et al., 2020; Chua et al., 2021). In order to explore the deeper association mechanism between the EMT and the prognosis of NSCLC, we compared the DEGs in the two groups and found that SFTA2 was significantly down-regulated in the C2 group. The overall expression of SFTA2 in lung cancer samples was lower in tumor tissues than that in normal lung tissues, and the low expression of SFTA2 was significantly related to the poor progression-free survival of NSCLC patients. Interestingly, Mittal et al. (2012) characterized SFTA2 as a novel secretory peptide highly expressed in the lung and is modulated by lipopolysaccharide. SFTA2 was also identified as prognostic genes with significantly correlation with the pathological stages of pancreatic ductal adenocarcinoma in an integrated transcriptome meta-analysis (Atay, 2020). So far, we have verified that SFTA2 gene could be used as an independent signature to predict the prognosis and TKIs resistance of lung cancer.

However, there still exist some limitations of this study. We did not include real-world cohort samples to study the comparison of EMT in tumor tissues and normal tissues, but we analyzed the differences between the EMT C1 and C2 groups and the corresponding prognosis through a large number of independent cohorts, revealing the significance of EMT in the prognosis of the patient.

## Conclusion

In conclusion, this study revealed the EMT process involved in the malignant progression of NSCLC. The construction of EMT clusters showed significant predictive value for NSCLC patients. In addition, we first identified the high expression of SFTA2 associated with better prognosis could serve as predictive biomarker for outcomes, dysregulation of TME, and immunotherapy response for NSCLC patients.

## Data Availability

The original contributions presented in the study are included in the article/Supplementary Material; further inquiries can be directed to the corresponding authors.
