# Supplementary figures and images for: Transcriptomic and immunologic implications of the epithelial–mesenchymal transition model reveal a novel role of SFTA2 in prognosis of non-small-cell lung carcinoma
Source: Front Genet. 2022 Aug 26;13:911801. doi: 10.3389/fgene.2022.911801 (PMC9458971; doi:10.3389/fgene.2022.911801)

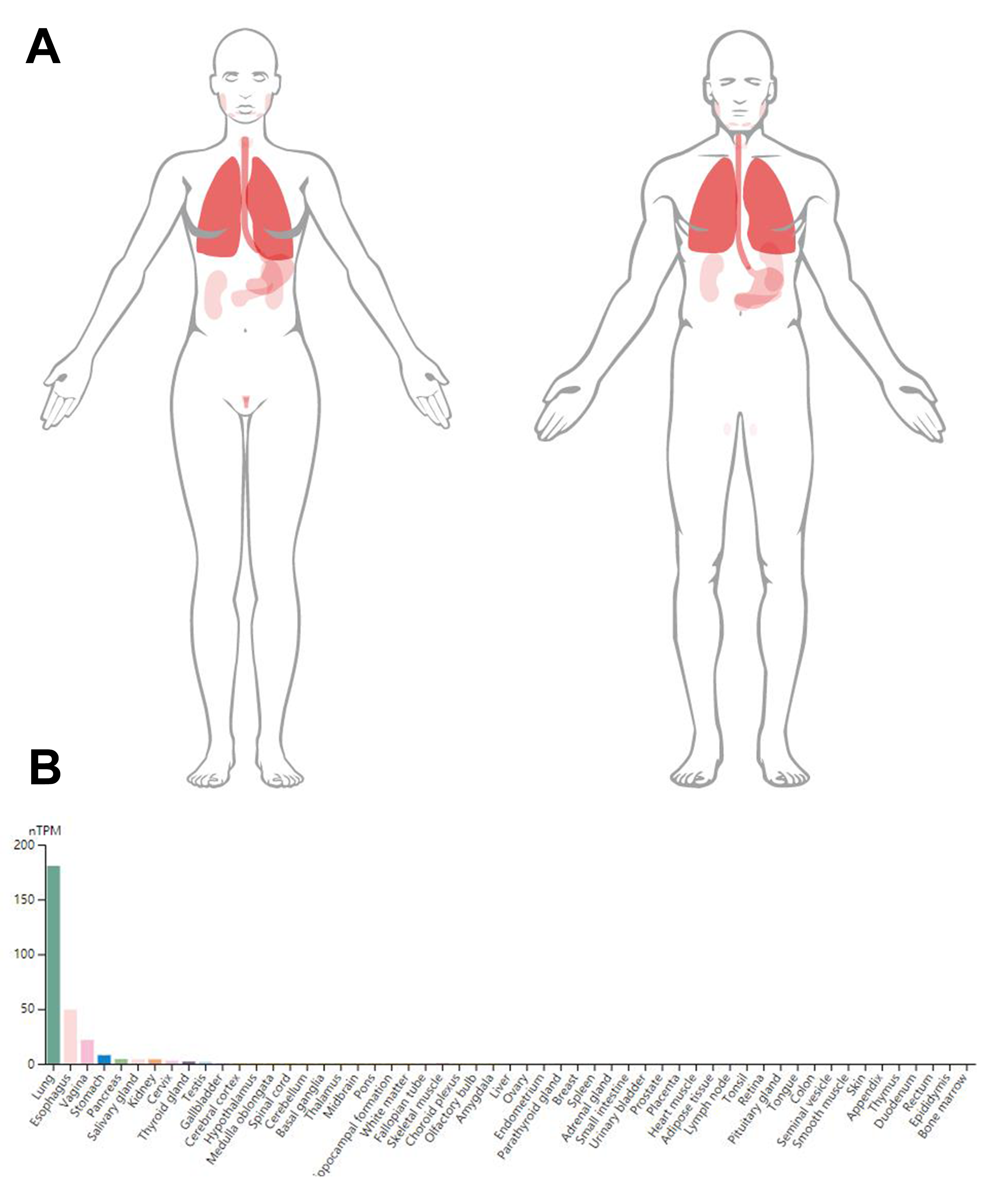

Supplement: Supplementary file 1 [file Image3.TIF]

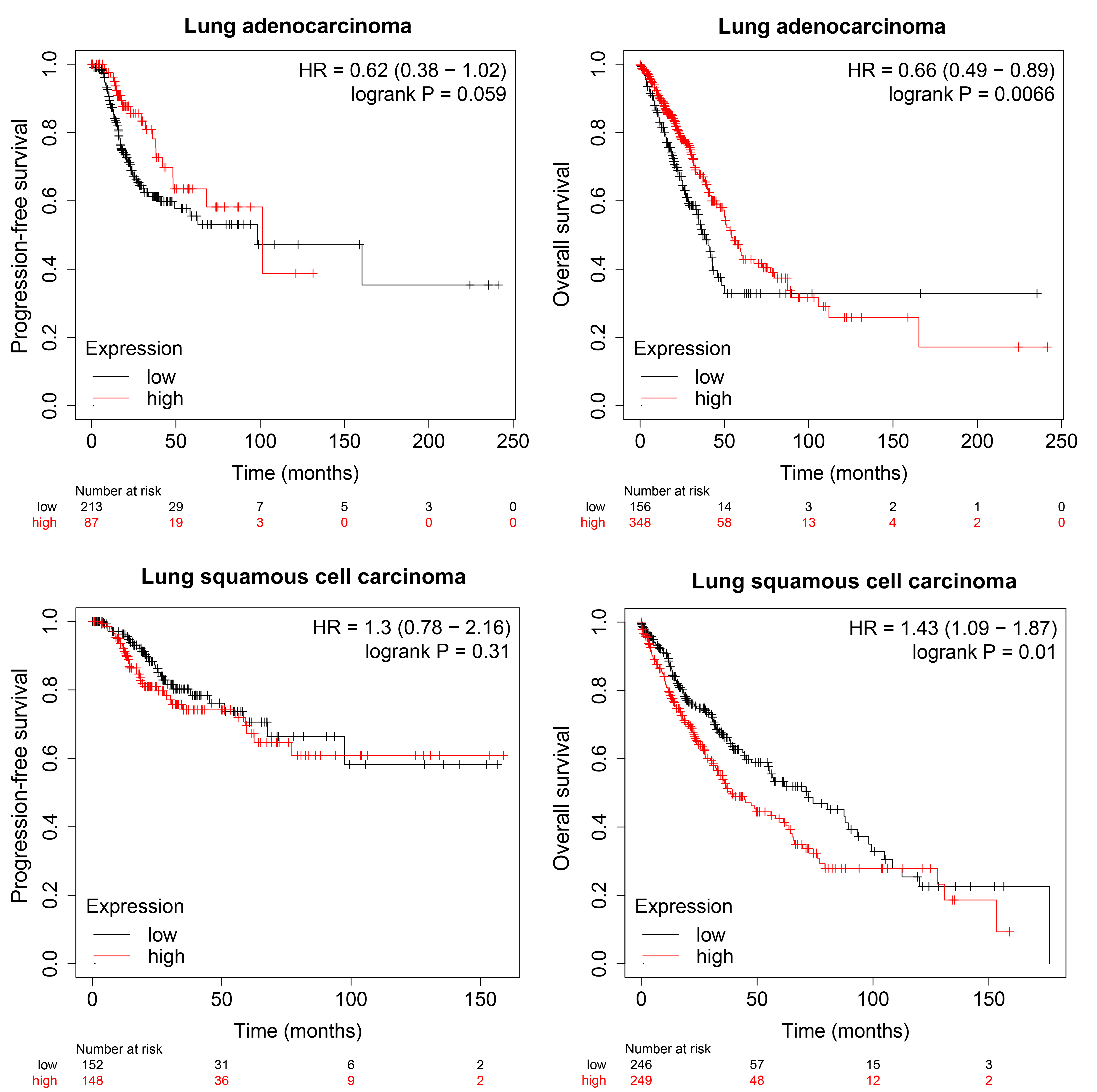

Supplement: Supplementary file 2 [file Image4.TIF]

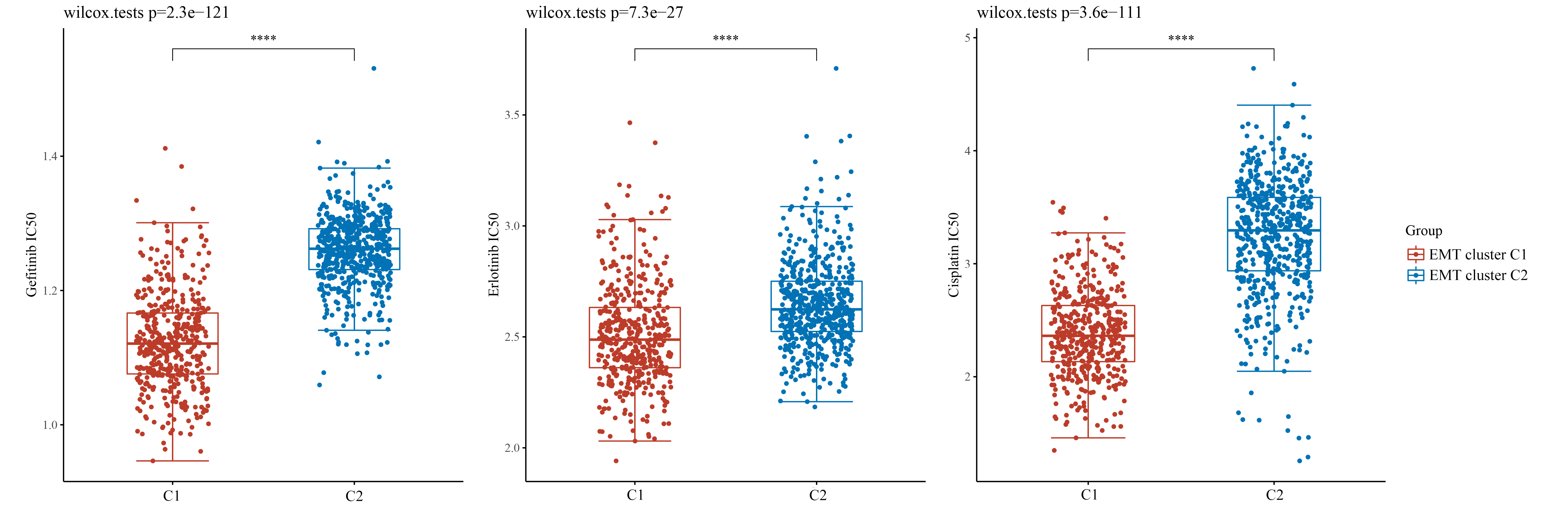

Supplement: Supplementary file 3 [file Image2.TIF]

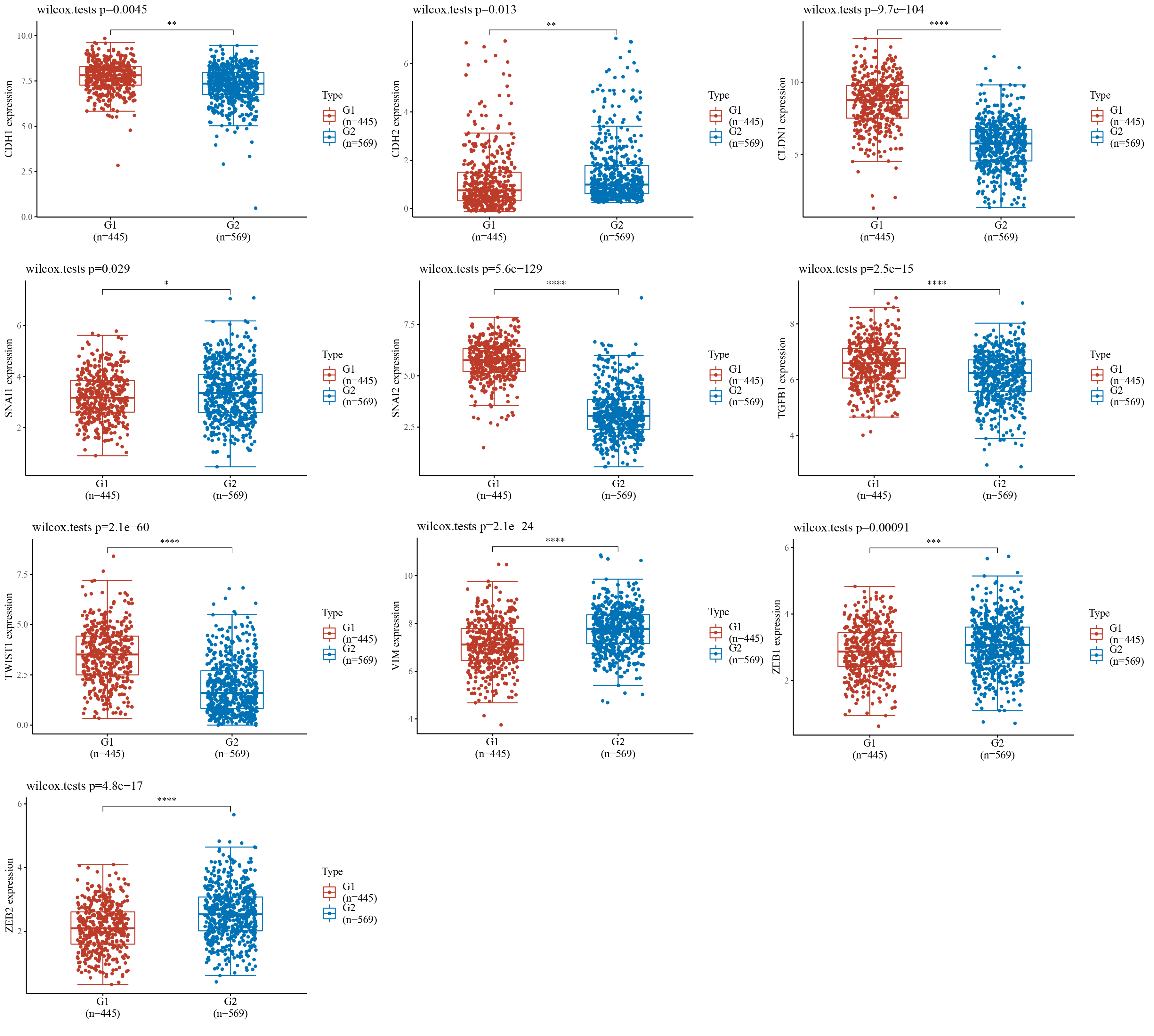

Supplement: Supplementary file 4 [file Image1.TIF]
